# Pulmonary hypertension alters blood flow distribution and impairs the hyperemic response in the rat diaphragm

**DOI:** 10.3389/fphys.2023.1281715

**Published:** 2023-12-22

**Authors:** Kiana M. Schulze, Andrew G. Horn, Ramona E. Weber, Bradley J. Behnke, David C. Poole, Timothy I. Musch

**Affiliations:** ^1^ Department of Kinesiology, Kansas State University, Manhattan, KS, United States; ^2^ Department of Anatomy and Physiology, Kansas State University, Manhattan, KS, United States

**Keywords:** perfusion, oxygen transport, vascular dysfunction, monocrotaline, respiratory muscle

## Abstract

Pulmonary hypertension (PH) is characterized by pulmonary vascular remodeling, respiratory muscle and cardiac impairments, and exercise intolerance. Specifically, impaired gas exchange increases work of the diaphragm; however, compromised contractile function precludes the diaphragm from meeting the increased metabolic demand of chronic hyperventilation in PH. Given that muscle contractile function is in part, dependent upon adequate blood flow ( $\dot{Q}$ ), diaphragmatic dysfunction may be predicated by an inability to match oxygen delivery with oxygen demand. We hypothesized that PH rats would demonstrate a decreased hyperemic response to contractions compared to healthy controls.

**Methods:** Sprague-Dawley rats were randomized into healthy (HC, n = 7) or PH (n = 7) groups. PH rats were administered monocrotaline (MCT) while HC rats received vehicle. Disease progression was monitored via echocardiography. Regional and total diaphragm blood flow and vascular conductance at baseline and during 3 min of electrically-stimulated contractions were determined using fluorescent microspheres.

**Results:** PH rats displayed morphometric and echocardiographic criteria for disease (i.e., acceleration time/ejection time, right ventricular hypertrophy). In all rats, total costal diaphragm $\dot{Q}$ increased during contractions and did not differ between groups. In HC rats, there was a greater increase in medial costal $\dot{Q}$ compared to PH rats (55% ± 3% vs. 44% ± 4%, *p* < 0.05), who demonstrated a redistribution of $\dot{Q}$ to the ventral costal region.

**Conclusion:** These findings support a redistribution of regional diaphragm perfusion and an impaired medial costal hyperemic response in PH, suggesting that PH alters diaphragm vascular function and oxygen delivery, providing a potential mechanism for PH-induced diaphragm contractile dysfunction.

## 1 Introduction

Pulmonary hypertension (PH) is a chronic disease characterized by elevated pulmonary arterial pressures and structural remodeling of the pulmonary vasculature resulting in dyspnea, cardiac dysfunction, and premature death. PH can occur idiopathically or as a comorbidity, however, all PH etiologies lower exercise tolerance and quality of life (Halank et al., 2013). The pathological changes in the pulmonary vasculature with PH can induce pulmonary congestion and poor gas exchange, which culminate in an increased ventilatory demand (Sun et al., 2003; Laveneziana et al., 2013) and consequently, increased work of the primary respiratory muscle, the diaphragm. Impaired pulmonary function and ventilatory insufficiency (Sun et al., 2001; Sun et al., 2003) largely contribute to dyspnea at rest and during exercise, therefore, understanding the consequences of heightened work of the diaphragm in PH are integral to improving exercise capacity and quality of life for PH patients. Previous investigations in PH patients (Meyer et al., 2005; Kabitz et al., 2007; Laveneziana et al., 2013; Breda et al., 2014; Spiesshoefer et al., 2019) and animal models of PH (de Man et al., 2011; Manders et al., 2012; Ahn et al., 2013; Himori et al., 2017) demonstrate diaphragm weakness and contractile dysfunction. Presently, the precise mechanisms responsible for PH-induced diaphragm dysfunction remain unknown.

It was initially believed that PH-induced systemic dysfunction occurred solely due to cardiopulmonary pathology. While central dysfunction undoubtedly plays a substantial role, it is becoming evident that pathological changes extend beyond these central manifestations, specifically in skeletal muscle. Impaired strength, oxygenation, and mitochondrial function in peripheral skeletal muscle (Bauer et al., 2007; Mainguy et al., 2010; Schulze et al., 2021; Schulze et al., 2022; Malenfant et al., 2015a; Malenfant et al., 2015b; Dimopoulos et al., 2013) independent of reduced cardiac output support the notion of vascular dysfunction beyond the pulmonary circulation. It has been suggested that diaphragm contractile dysfunction precedes central hemodynamic compromise (Kosmas et al., 2022), however, the effects of PH on bulk and regional diaphragm perfusion have not been investigated directly. In order to sustain diaphragmatic contractile function, there must be a tight coupling of O_2_ delivery ( $\dot{Q}$ O_2_)-to-O_2_ demand ( $\dot{V}$ O_2_). Given that $\dot{V}$ O_2_ α $\dot{Q}$ O_2_ *x* PO_2_, inadequate $\dot{Q}$ O_2_ to maintain $\dot{V}$ O_2_ will necessitate a lower intramyocyte PO_2_, possibly inducing metabolic perturbations which hasten diaphragm muscle fatigue (Hogan et al., 1992; Poole et al., 1997; Poole et al., 2001; Davis et al., 2012). Whilst the diaphragm has properties that distinguish it from other skeletal muscle (e.g., continually active, high oxidative and vasodilatory capacities (Poole et al., 2000), countercurrent capillary flow (Kindig and Poole, 1998)), blood flow dysregulation in the hyperactive PH diaphragm (Laveneziana et al., 2013) could nevertheless result in local areas of hypoxia, which would thereby play a pivotal role in the increased oxidative stress (Himori et al., 2017) and decreased force generation (de Man et al., 2011) associated with PH-induced diaphragm dysfunction. Peripheral endothelial dysfunction has been demonstrated in PH (Wolff et al., 2007; Peled et al., 2008) and, if present in the diaphragm, would reduce the hyperemic response, exacerbating exercise intolerance and providing a putative mechanism for diaphragmatic contractile impairments and dyspnea in PH.

Therefore, the overall objective of this study was to investigate the effects of monocrotaline (MCT)-induced PH on bulk and regional diaphragmatic blood flow and vascular conductance. We tested the hypotheses that in MCT rats with moderate severity PH *versus* healthy control there will be: 1) lower diaphragm vascular conductance and blood flow, and 2) a decreased diaphragm hyperemic response to 1 Hz contractions. Additionally, answering this question will provide insights to the mechanistic bases for the diaphragm dysfunction associated with PH.

## 2 Materials and methods

### 2.1 Animals

Fourteen female Sprague-Dawley rats (4–6 months old) were obtained from Charles River Laboratories (Boston, MA, USA) for this investigation. The greater prevalence of PH in females (Badesch et al., 2010; Hester et al., 2019), in combination with the paucity of literature examining females with PH, provides support for our use of female rats herein. Rats were randomized into healthy control (HC, n = 7) and monocrotaline (MCT)-induced pulmonary hypertension animals (MCT, n = 7). The Sprague-Dawley rat was chosen due to the similar properties (e.g., anatomical and physiological) of its diaphragm to the human diaphragm (Metzger et al., 1985; Mizuno, 1991; Poole et al., 1997). The diaphragm is comprised of two distinct muscle regions: the costal (ventral, medial, and dorsal) and the crural diaphragm. We elected to focus on the medial costal region. Given its anatomical position and fiber orientation, the medial costal diaphragm sustains the highest proportion of ventilatory work (Poole et al., 1997) and receives the highest mass specific blood flow within the costal diaphragm (Davis et al., 2012; Horn et al., 2020; Horn et al., 2022; Poole et al., 1997; Poole et al., 2000), therefore serving as the primary driver for inspiration. All procedures were approved by the Kansas State University Institutional Animal Care and Use Committee and complied with the National Institutes of Health Guide for the Care and Use of Laboratory Animals. Upon arrival, animals were housed and maintained in a temperature-controlled (23°C ± 2°C) room with a 12:12-h light-dark cycle with water and rat chow provided *ad libitum*.

### 2.2 Monocrotaline-induced pulmonary hypertension

Pulmonary hypertension (PH) was induced using monocrotaline alkaloid (MCT; 50 mg/kg) administered via a single intraperitoneal injection which has been shown to induce progressive PH (Gomez-Arroyo et al., 2012; Schulze et al., 2021; Schulze et al., 2022). MCT (BOC Sciences; Shirley, NY) was dissolved in a solution containing 50% sterile saline and 50% 200-proof ethanol at room temperature and a total of 0.5 mL of fluid was injected into each rat in the experimental group while HC rats received 0.5 mL of vehicle. While maintained on 1%–2% isoflurane, injections were given immediately following pre-injection echoes (see *Echocardiography*) with a 27 G needle in the lower right quadrant of the abdomen with care taken to avoid internal organs. Importantly, characteristics of PH (i.e., elevations in pulmonary artery pressures, remodeling of small pulmonary arteries, and right ventricular (RV) hypertrophy) occur 3–4 weeks following injection, prior to the development of overt heart failure (Gomez-Arroyo et al., 2012). Animals were monitored weekly via echocardiography along with measures of body weight and body condition scoring.

### 2.3 Echocardiography

Transthoracic echocardiography was performed using a commercially available system (Logiq S8; GE Health Care, Milwaukee, WI) with a 13 MHz linear transducer (L4-12t) prior to MCT or saline injection. Subsequent transthoracic echocardiography was performed weekly following injection to monitor disease progression. All comparisons were made between the pre-injection measurement and final measurement taken just prior to experimentation. Animals were initially anaesthetized by inhalation of a 5% isofluorane-O_2_ mixture and maintained on ≤2.0% isofluorane-O_2_ (Butler Health Supply, Dublin, OH). Core temperature was maintained at ∼37°C, measured via rectal thermometer. 2-D and M-mode images of the left ventricle (LV) were obtained from the parasternal short axis window and analyzed for end-systolic/diastolic volumes, stroke volume, and ejection fraction as previously described (Craig et al., 2019). RV measurements were taken at the level of the aortic valve, just proximal to the pulmonary valve, in the short axis view. Pulsed Doppler ultrasound was used to assess pulmonary artery ejection time (ET), acceleration time (AT), and peak velocity, and the ratio of AT to ET (AT/ET) was calculated and used as a clinical parameter for the confirmation of PH (AT/ET < 0.3) (Jones et al., 2002). Upon verification of PH, (average 23 days post-injection) the following experiments were performed.

### 2.4 Surgical preparation

All surgical procedures were performed using aseptic techniques. Rats were initially anesthetized with a 5% isoflurane-O_2_ mixture for 5 min (isoflurane vaporizer; Harvard Apparatus, Cambridge, MA) and subsequently maintained on 3% isoflurane-O_2_. Body temperature was maintained at 37°C ± 1°C (via rectal thermometer) by use of a water-recirculating heating blanket. An incision was made on the ventral side of the neck and the left carotid artery was isolated and cannulated with PE-10 connected to PE-50 (Intra-Medic polyethylene tubing; Clay Adams Brand, Becton, Dickinson, Sparks, MD) for measurements of mean arterial pressure (MAP; Digi-Med BPA, Micro-Med Inc., Louisville, KY) and infusion of fluorescent microspheres (*see below*). A second catheter (PE-10 connected to PE-50) was inserted into the caudal artery for the infusion of pentobarbital sodium anesthesia (50 mg/mL) and reference sampling for blood flow determination. Rats were then transitioned to pentobarbital sodium anesthesia (20 mg/kg body wt) given intra-arterially while concentrations of isoflurane were decreased and subsequently discontinued over ∼30 min. The level of anesthesia was regularly monitored via toe pinch and palpebral reflex, with pentobarbital anesthesia supplemented (3.5–7.0 mg/kg) as necessary.

### 2.5 Diaphragm contractions

Following carotid and tail artery catheterization, animals were tracheotomized and mechanically ventilated (Kent Scientific PhysioSuite, Torrington, CT). The diaphragm was exposed via laparotomy as previously described (Poole et al., 1995; Geer et al., 2002; Davis et al., 2012). Following diaphragm exposure, stainless steel electrodes were sutured to the inferior aspect of the right ventral costal (cathode) and the right dorsal costal (anode) diaphragm. Prior to contractions, fluorescent microspheres were infused (*described below*) to assess baseline diaphragmatic blood flow in the inactive diaphragm. Thereafter, electrically stimulated twitch contractions were induced at 1 Hz (3–6 V, 2-ms pulse duration) with a Grass S88 stimulator (Quincy, MA) for 180s. This stimulation protocol elicits diaphragm contractions at a frequency similar to that of a spontaneously breathing animal (Geer et al., 2002). Blood flow was measured via fluorescent microspheres (*described below*) at 180s of stimulation to assess the contracting steady-state diaphragm hyperemia.

### 2.6 Fluorescent microsphere injection

The fluorescent microsphere technique, as previously described (Deveci and Eggington, 1999; Musch et al., 1986), was used to quantify tissue blood flow in each experimental group. Fluorescent microspheres were infused at two different time points: 1) baseline (10 min following laparotomy) and 2) at 180s of electrically induced contractions. For each measure, a reference blood sample was taken from the caudal artery catheter, using a Harvard withdrawal pump (model 907, Cambridge, MA) that was initiated 30s prior to microsphere infusion at a withdrawal rate of 0.25 mL/min and 2.0–2.5 × 10^5^ fluorescent microspheres (colors: red, scarlet, or blue-green; 15.5 μm diameter, Invitrogen FluoSpheres, Carlsbad, CA) were infused into the aortic arch via the carotid artery catheter. Adequate mixing of microspheres was determined by <20% difference in left and right kidney or left and right soleus muscle blood flows at each time point. Following the final microsphere infusion, rats were euthanized with pentobarbital sodium overdose (>50 mg/kg I.A.) followed by cardiac excision. Thereafter, tissues (diaphragm, soleus, intercostal muscles, and kidneys) were harvested, weighed, and placed in 15 mL screw cap polypropylene conical tubes and then placed in a −80°C freezer for later blood flow analysis. Using the template previously described (Horn et al., 2020; Horn et al., 2022; Poole et al., 1997), the costal diaphragm was sectioned into ventral, medial, and dorsal portions to determine regional distribution of diaphragmatic blood flow, whereas the sum of these portions was used to calculate total costal diaphragm blood flow.

### 2.7 Calculation of blood flow and vascular resistance

The fluorescent microsphere assay was performed as previously described (Deveci and Eggington, 1999; Horn et al., 2020; Horn et al., 2022). Tissue blood flow was calculated as follows (Deveci and Eggington, 1999):

$$\dot{Q}=\left[\left(A_{t}\;/\;A_{b}\right)\;x\;\left(s/w\right)\right]x\;100$$

where $\dot{Q}$ is blood flow (ml/min/100g), *A*_*t*_ is the individual sample intensity, *A*_*b*_ is the reference blood sample intensity, *s* is the withdrawal rate (0.25 mL/min) of the reference blood sample, and *w* is the tissue weight (g). Vascular conductance was calculated as:

$$VC=\;\dot{Q}/\;MAP$$

where *VC* is vascular conductance (ml/mmHg/min/100g), $\dot{Q}$ is blood flow (ml/min/100g), and *MAP* is the average mean arterial pressure (mmHg), recorded immediately before and after microsphere infusion. These assays utilized the entire diaphragm and therefore precluded tissue availability for corollary measurements.

### 2.8 Postmortem measurements

The RV, LV, and lungs were dissected and weighed following euthanasia. RV hypertrophy was measured via the Fulton index: RV weight (mg)/LV + septum (S) weight (mg) (Fulton et al., 1952). RV hypertrophy was also expressed as RV/body weight.

### 2.9 Data analysis

Data were analyzed using GraphPad Prism9 (GraphPad Software, San Deigo, CA). Student paired (within animal) and independent two-sample (between HC and MCT) t-tests were utilized to determine differences in echocardiographic and morphometric measurements. Body mass (g), diaphragm mass (mg), and diaphragm:body mass ratio were analyzed using a one-way ANOVA. Tissue blood flows were analyzed with a mixed-effects model. A Grubb’s outlier test was performed on all data, and 1 outlier was identified and removed from the respective data subset when detected. Post-hoc analyses were performed using a Tukey’s test. All data are presented as means ± SE. Significance was established at *p* < 0.05.

## 3 Results

### 3.1 Morphometric and echocardiographic measurements

All MCT rats displayed characteristics consistent with PH including a decreased AT/ET ratio, RV hypertrophy (RV/LV + S), and an elevated lung weight compared to HC animals (Tables 1,2). Total diaphragm weight did not differ significantly between groups (Table 1). There were no differences in left ventricular ejection fraction, fractional shortening, or stroke volume between HC and MCT rats (Table 2; all *p* > 0.05]. Furthermore, heart rate, cardiac output, and cardiac index did not differ between groups (Table 3), so we do not believe cardiac factors associated with blood flow to be a limitation to interpretation of the findings herein. MAP was not different between groups, or when comparing baseline to diaphragm contractions (Table 3). Additionally, there were no differences between groups in regional diaphragm masses, nor in total costal diaphragm mass [all *p* > 0.05].

**TABLE 1:** Morphometric measurements. Data are means ± SE; *n,* number of rats*; n* = 7 for all; PH, pulmonary hypertension; RV, right ventricle; LV, left ventricle; LV + S, LV + septum; # *p* < 0.05 vs. healthy control.

	Healthy control	Monocrotaline PH
Body weight (g)	297 ± 6	276 ± 7 #
Diaphragm weight (mg)	944 ± 67	938 ± 42
RV weight (mg)	174 ± 10	197 ± 11
LV weight (mg)	681 ± 24	573 ± 19 #
Lung weight (mg)	1188 ± 26	1712 ± 126 #
RV/LV + S (mg/mg)	0.26 ± 0.02	0.37 ± 0.02 #
Diaphragm/body weight (mg/g)	3.18 ± 0.17	3.40 ± 0.08
RV/body weight (mg/g)	0.59 ± 0.03	0.72 ± 0.06 #
LV/body weight (mg/g)	2.30 ± 0.09	2.09 ± 0.08
Lung/body weight (mg/g)	4.0 ± 0.1	6.3 ± 0.5 #

**TABLE 2:** Echocardiographic measurements. Data are means ± SE; *n,* number of rats*; n* = 7 for all; PH, pulmonary hypertension; AT, acceleration time; ET, ejection time; #*p* < 0.05 vs. all other conditions.

	Healthy control		Monocrotaline PH	
	Pre	Post	Pre	Post
Acceleration time (ms)	30 ± 2	33 ± 4	29 ± 2	19 ± 1 #
Ejection time (ms)	84 ± 2	85 ± 2	82 ± 3	81 ± 3
AT/ET	0.36 ± 0.02	0.39 ± 0.04	0.35 ± 0.02	0.23 ± 0.01 #
Ejection fraction (%)	84 ± 2	86 ± 3	85 ± 2	86 ± 4
Stroke volume (mL)	0.48 ± 0.05	0.53 ± 0.02	0.53 ± 0.04	0.52 ± 0.05
Fractional shortening (%)	48 ± 3	52 ± 4	50 ± 2	53 ± 5

**TABLE 3:** Central hemodynamics. Data are means ± SE; *n,* number of rats*; n* = 7 for all; PH, pulmonary hypertension.

	Healthy control	Monocrotaline PH
Heart rate (bpm)	353 ± 11	375 ± 12
Cardiac output (mL/min)	188 ± 11	196 ± 17
Stroke index (mL/kg)	1.8 ± 0.1	1.9 ± 0.2
Cardiac index (mL/min/kg)	642 ± 44	712 ± 61
Mean arterial pressure (mmHg)	119 ± 2	114 ± 3

### 3.2 Blood flow

**FIGURE 1:**
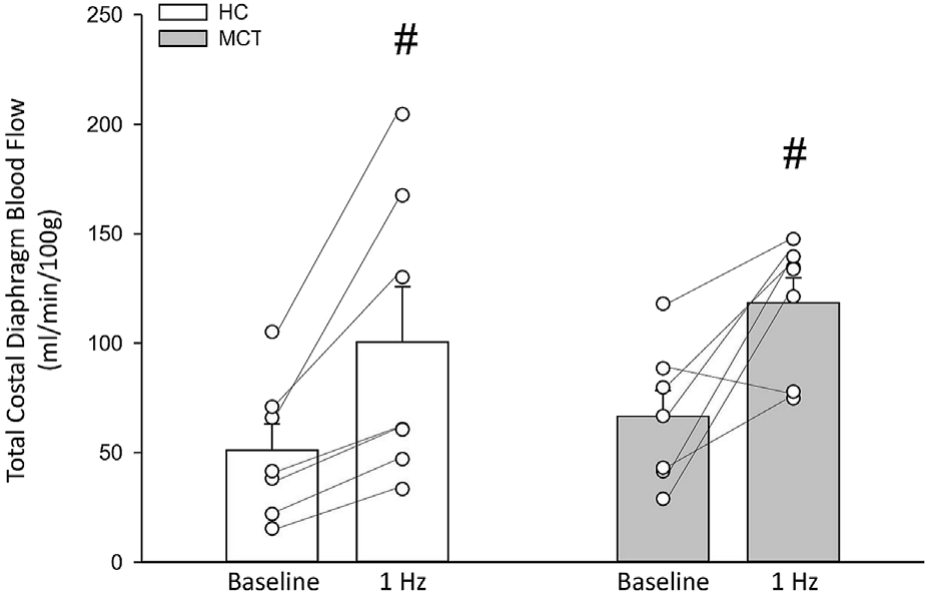
Total costal diaphragm blood flow (ml/min/100 g) in healthy control (HC; n = 7) and monocrotaline-induced pulmonary hypertension (MCT; n = 7) animals at baseline and during steady-state 1 Hz contractions. #Significant (*p* < 0.05) *versus* baseline.

**FIGURE 2:**
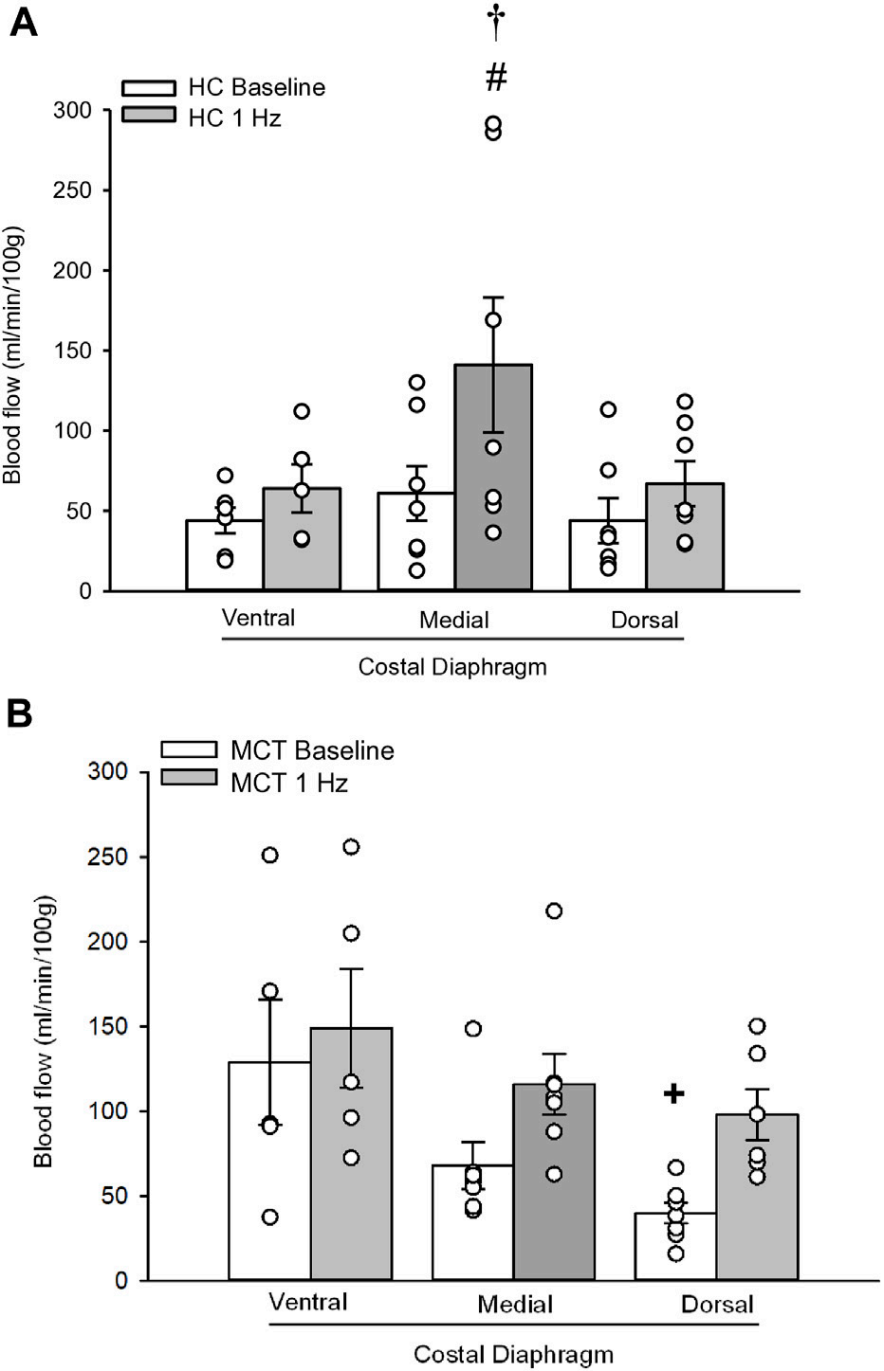
Regional diaphragm blood flow (ml/min/100 g) at baseline and during steady-state 1 Hz contractions in healthy control (HC; n = 7) **(A)** and monocrotaline-induced pulmonary hypertension (MCT; n = 7) **(B)** animals. #Significant (*p* < 0.05) *versus* baseline within diaphragm region. †Significant (*p* < 0.05) *versus* other diaphragm regions at 1 Hz +Significant (*p* < 0.05) *versus* ventral costal baseline.

**FIGURE 3:**
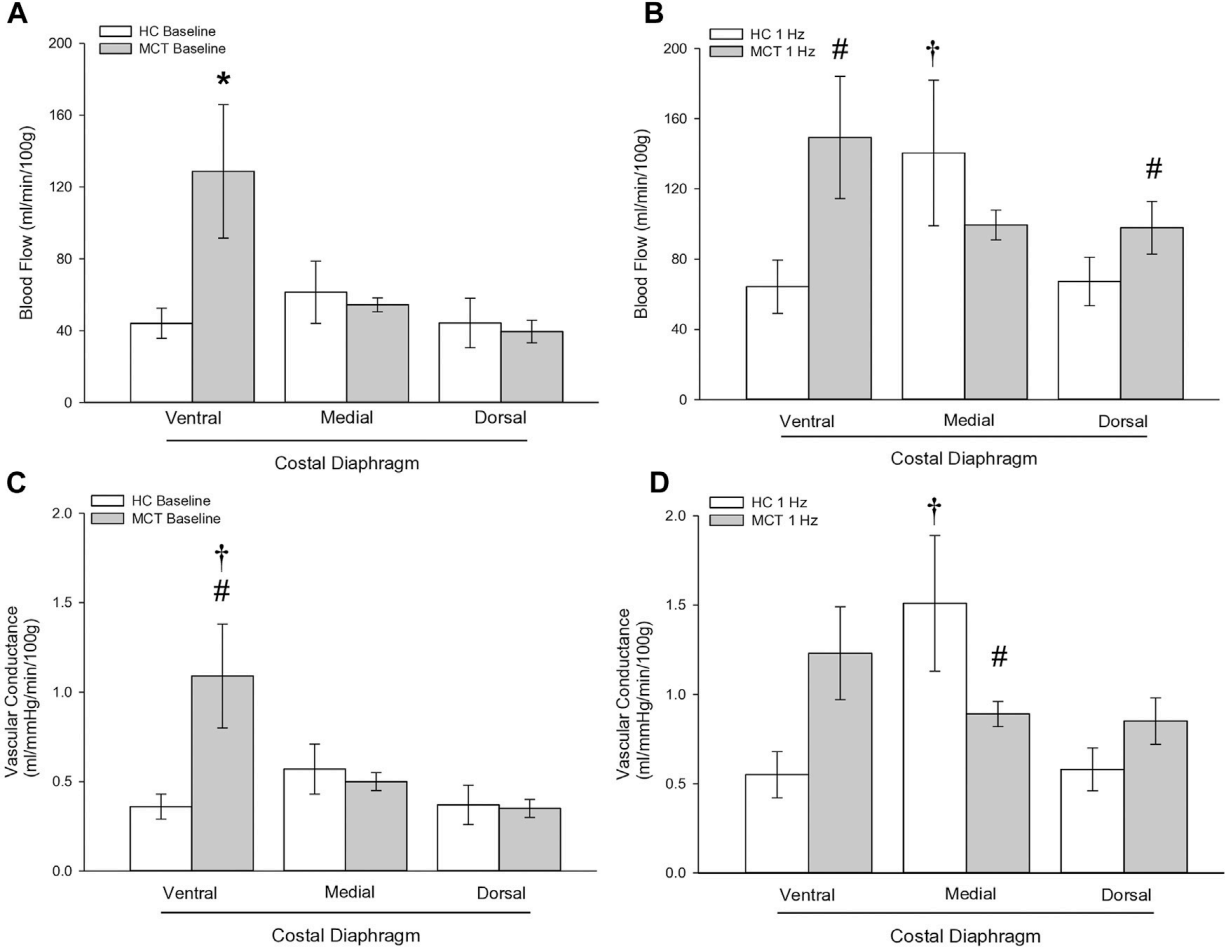
Regional diaphragm blood flow at baseline **(A)** and during steady-state 1 Hz contractions **(B)** and vascular conductance (ml/mmHg/min/100 g) at baseline **(C)** and during steady-state 1 Hz contractions **(D)** in healthy control (HC; n = 7) and monocrotaline-induced pulmonary hypertension (MCT; n = 7) animals. *Significant (*p* < 0.05) *versus* all other bars (Figure 3A). #Significant (*p* < 0.05) *versus* HC within diaphragm region. †Significant (*p* < 0.05) *versus* other diaphragm regions within group (HC or MCT).

**FIGURE 4:**
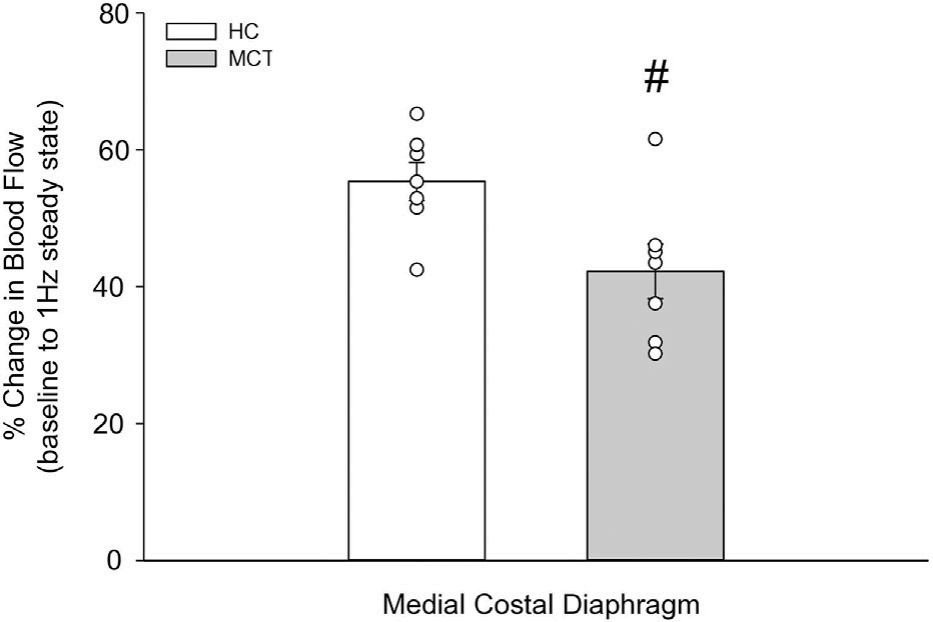
The change in medial costal diaphragm blood flow from baseline to steady-state 1 Hz contractions in healthy control (HC; n = 7) and monocrotaline-induced pulmonary hypertension (MCT; n = 7) animals. #Significant (*p* < 0.05) *versus* HC.

At baseline (i.e., in the inactive diaphragm), total costal diaphragm blood flow was not different between groups (Figure 1). Despite this, MCT rats had a higher baseline ventral costal blood flow compared to HC and all other costal regions of the diaphragm in MCT and HC rats (Figures 2 and 3A). No regional differences were present between costal portions of the diaphragm in HC rats at baseline (Figure 2A and Figure 3A). In response to 1 Hz contractions, total costal diaphragm blood flow increased in both HC and MCT animals (Figure 1). In HC rats, 1 Hz contractions elicited the greatest blood flow increase to the medial costal diaphragm (Figure 2A); whereas in MCT animals, the ventral costal portion received the highest baseline and contracting blood flow, and the dorsal costal had the largest increase in blood flow compared to baseline (Figure 2B). Therefore, the hyperemic response of the medial costal diaphragm was greater in HC rats, increasing 55% ± 3% compared to baseline *versus* only 44% ± 4% in MCT rats (Figure 2A and Figure 4; *p* = 0.04), while the total costal diaphragm hyperemic response was not different between groups (HC, 49% ± 4% vs. MCT, 41% ± 12%, *p* = 0.55). Kidney blood flows in all conditions fell within the 400–600 mL/min/100 g range expected based on previous investigations from our laboratory (Horn et al., 2020; Horn et al., 2022; Colburn et al., 2020). There were no differences between group or condition in blood flow to the kidneys, soleus, or intercostal muscles (Table 4; all *p* > 0.05).

**TABLE 4:** Blood flow. Data are means ± SE; *n,* number of rats*; n* = 7 for all; PH, pulmonary hypertension.

	Healthy control		Monocrotaline PH	
	Baseline	1 Hz	Baseline	1 Hz
Kidney	431 ± 46	457 ± 56	505 ± 70	594 ± 94
Soleus	48 ± 14	45 ± 15	46 ± 11	32 ± 7
Intercostal	38 ± 16	31 ± 8	21 ± 4	29 ± 10

### 3.3 Vascular conductance

Total costal diaphragm vascular conductance was not different between HC and MCT rats at baseline (0.38 ± 0.1 vs. 0.46 ± 0.1 mL/mmHg/min/100g; *p* = 0.5) or during 1 Hz steady-state contractions (0.83 ± 0.18 vs. 0.87 ± 0.08 mL/mmHg/min/100g; *p* = 0.8). At baseline, vascular conductance was highest in the ventral costal portion of the diaphragm in MCT rats (Figure 3C). During contractions, vascular conductance was highest in the medial costal portion for HC rats, and the ventral costal portion for MCT rats (Figure 3D). Vascular conductance did not differ between groups in the kidneys, soleus, or intercostals (all *p* > 0.05).

## 4 Discussion

This is the first investigation to our knowledge to demonstrate that PH alters regional diaphragm blood flow distribution and impacts the ability of the diaphragm to augment blood flow with contractile activity. Specifically, PH induced a greater distribution of diaphragm blood flow to the ventral costal diaphragm, a region that in health contributes proportionally less to the work of breathing than the medial and dorsal costal diaphragm. Further, the blunted diaphragm hyperemic response to 1 Hz contractions suggests that PH compromises diaphragm vascular function, potentially via a decreased vasodilatory responsiveness and/or increased tonic vasoconstriction of the diaphragm resistance vasculature. These findings in a female rat model of PH raise the possibility that the pathophysiological consequences of PH extend into the respiratory muscle vasculature and provide a putative mechanism for the diaphragm contractile dysfunction associated with PH.

### 4.1 Monocrotaline-induced PH

This investigation utilized the MCT model of PH at a dose demonstrated to elicit characteristics consistent with the human pathology within 3–4 weeks including medial wall thickening of the pulmonary arteries, elevated right ventricular systolic pressure, pulmonary congestion, and RV hypertrophy (Gomez Arroyo et al., 2012; Nogueira-Ferreria et al., 2015; Schulze et al., 2021; Schulze et al., 2022). RV hypertrophy is commonly determined using the Fulton Index (RV/LV + S) or RV/body weight. In this investigation, MCT animals were smaller; however, RV mass was not different, demonstrating a compensatory RV hypertrophy in response to the higher expected afterload. Additionally, PH was confirmed in these rats using clinically established echocardiographic measurements (i.e., AT/ET) (Jones et al., 2002; Kitabatake et al., 1983). Lower AT/ET has been demonstrated by Kato et al. to be associated with increased RV myocyte cross sectional area and elevated RV/body weight (Kato et al., 2003). Importantly, this model induces moderate PH, indicating that the alterations in diaphragm blood flow presented herein occur prior to, and independent of, distinct heart failure as indicated by lack of changes in LV ejection fraction (Table 2).

### 4.2 Diaphragmatic blood flow and vascular conductance

Contrary to our hypothesis, bulk diaphragm blood flow (Figure 1) and vascular conductance were not different in MCT rats *versus* HC. Given the heterogenous distribution of blood flow in the diaphragm (Brancatisano et al., 1991; Davis et al., 2012; Poole et al., 1997; Sexton and Poole, 1995; Horn et al., 2020), altered distribution of regional diaphragm blood flow may represent significant changes in the diaphragm vasculature with PH that are masked by only investigating whole diaphragm hemodynamics. In the healthy rodent (i.e., rat), the medial costal diaphragm sustains the greatest proportion of diaphragm inspiratory work and therefore commands the highest blood flow (Poole et al., 1997; Sexton and Poole, 1995). The diaphragm contractions protocol herein has been used previously in our laboratory and is expected to produce a stimulus akin to spontaneous breathing (Geer et al., 2002; Davis et al., 2012). Diaphragm blood flow data in the current investigation are consistent with spontaneously breathing animals (Horn et al., 2020; Horn et al., 2022). In HC animals, medial costal diaphragm blood flow was augmented from baseline to steady-state contractions as expected (Figure 2A); however, in MCT animals there was no significant increase in medial costal diaphragm perfusion during steady-state contractions (Figure 2B). Further, medial costal diaphragm vascular conductance during contractions was lower in MCT rats compared to HC (Figure 3D). These data support that PH impairs the hyperemic response, specifically in the medial costal diaphragm (Figure 4). Lower medial costal vascular conductance and the reduced ability to augment blood flow with contractile activity suggest that PH induces vascular dysfunction in the diaphragm. Such vascular impairments in the diaphragm with PH may be due to a reduced vasodilatory responsiveness, enhanced intrinsic vascular tone, endothelial or vascular smooth muscle signaling mechanisms, changes in neurovascular coupling, and/or structural alterations in the diaphragm resistance vasculature that manifest throughout disease progression, though this remains to be determined.

Previous work suggests that diaphragm contractile dysfunction is predicated by an inability to match oxygen delivery to oxygen demand (i.e., upstream vascular dysfunction) (Supinski, 1988; de Man et al., 2011; Manders et al., 2012). Our data demonstrate reduced blood flow, and therefore oxygen delivery, to the medial costal diaphragm. Given that blood flow distribution and functional hyperemia is determined by vascular control mechanisms, the blunted medial costal hyperemic response seen with PH is likely due in large part to impaired resistance vessel (i.e., arterioles) vasodilation. Arteriolar dysfunction has been shown across a range of pathological conditions (i.e., aging, hypertension, heart failure, prolonged mechanical ventilation) in several vascular beds [for rev. Vanhoutte et al., 2017; Poole et al., 2021], including skeletal muscle (Muller-Delp et al., 2002) and the diaphragm (Horn et al., 2019). Impaired endothelial function has been demonstrated in pulmonary arteries (Mathew et al., 1995; Christou et al., 2018), coronary arterioles (Kajiya et al., 2007), and large systemic arteries (Wolff et al., 2007; Peled et al., 2008) with PH. However, there is a paucity of data regarding diaphragm vascular function. In PH, dysregulation of the nitric oxide (NO) pathway and production of excess reactive oxygen species (ROS) (Giaid and Saleh, 1995; Humbert et al., 2004; Hampl and Herget, 2000) may act in concert to reduce endothelium-dependent vasodilation [Poole et al., 1997). Further, the increased levels of endothelin-1 (Rubens et al., 2001) and Angiotensin II (Kumpers et al., 2010) in PH would increase tonic vasoconstriction and blunt NO-mediated vasodilation (Mathew et al., 1995). The degree to which these markers are upregulated is associated with disease severity. In the present study where animals presented with moderate PH preceding heart failure, the extent of the impact of these vasoconstrictors is not known. Additionally, in the hyperactive diaphragm with PH, $\dot{Q}$ O_2_-to- $\dot{V}$ O_2_ mismatch during increases in metabolic demand could result in local areas of hypoxia (i.e., medial costal) that can promote further ROS generation, thereby damaging the vasculature (Marzetti et al., 2013) and potentially compromising contractile function (Hogan et al., 1992; Hogan et al., 1994; Himori et al., 2017). Potential structural alterations in the diaphragm vasculature with PH have not been investigated but may play a role in the impaired hyperemic response demonstrated herein. Prolonged arteriolar vasoconstriction can induce vascular remodeling in as little as 4 h (Martinez Lemus et al., 2004). Thus, it is possible that the sympathetic hyperactivity, inflammation, and oxidative stress associated with PH could induce vascular remodeling beyond the pulmonary circulation, thereby limiting the necessary increases in medial costal blood flow with contractile activity (Dorfmuller et al., 2003).

Interestingly, ventral costal diaphragm perfusion was higher in MCT rats compared to control both at baseline (Figure 3A) and during 1 Hz steady-state contractions [Figure 3B), suggesting that PH results in altered blood flow distribution. Considering adequate blood flow and thus resistance vessel dilation constitute a major determinant of contractile function (Supinski, 1988; Supinski et al., 1993), an inability to meet respiratory muscle oxygen demand would hasten diaphragm fatigue. Therefore, maintenance of a heightened metabolic demand in the chronically hyperactive diaphragm with PH (Laveneziana et al., 2013) may require recruitment of regions that do not contribute as significantly in health. Such a redistribution of blood flow within the diaphragm may actually serve to better match $\dot{Q}$ O_2_ to $\dot{V}$ O_2_, although, whether this regional redistribution has a positive or negative effect on local tissue oxygenation, as mentioned above, remains to be determined. Given the physical structure of the ventral costal region of the diaphragm (Poole et al., 1997), greater blood flow to this region at the expense of that to the medial costal region would be expected to reduce overall diaphragmatic contractile force and compromise ventilatory efficiency, thereby serving as a potential mechanism for the diaphragm contractile dysfunction associated with PH.

### 4.3 Absence of diaphragm atrophy

Previous investigations in male murine models of PH (Ahn et al., 2013; de Man et al., 2011) demonstrate diaphragm atrophy, however, these studies differ in MCT dosage and time course for PH development (6–8 weeks). In contrast, our MCT rats did not display diaphragm atrophy. While we did not assess cross sectional area of muscle fibers, diaphragm mass and diaphragm mass:body weight were not different between groups (Table 1). The MCT rats in the current investigation are studied prior to development of RV failure in order to assess moderate PH. It is possible that PH-induced diaphragm atrophy is the consequence of late-stage decompensation resulting from the inability to match $\dot{Q}$ O_2_ to $\dot{V}$ O_2_, thereby impairing contractile function, generating excess ROS, upregulating markers of atrophy, and inducing eventual diaphragmatic failure.

### 4.4 Experimental considerations

The present investigation focused on moderate PH preceding RV failure. A longer time course following MCT injection would allow for development of a more severe form of PH that may elicit diaphragm atrophy not seen herein. Future directions may usefully assess $\dot{Q}$ O_2_-to- $\dot{V}$ O_2_ matching in the diaphragm, regional tissue oxygenation, and use histological techniques to determine molecular/structural diaphragmatic or vascular alterations, as well as the time course for development of diaphragm atrophy, blood flow alterations, and contractile dysfunction in this model. Additionally, resting and exercising diaphragm blood flows in the conscious rat could provide insights into increased ventilatory demand, dyspnea, and the exercise intolerance seen with PH. The present investigation used only female rats, thus all findings should be regarded in the context of MCT-induced PH in the female rat. However, available evidence does not support that sex or stage of the estrus cycle impact diaphragm blood flow (Smith et al., 2017). Furthermore, because females display a higher incidence and greater prevalence of PH, there is a pressing imperative to examine females in PH-focused research (Badesch et al., 2010; Hester et al., 2019). Future studies may include assessment of the effect of estrogen on the specific diaphragm perfusion measurements herein by inclusion of both male and ovariectomized female rats. A more in-depth discussion of the MCT-PH model in female rats and the necessity to focus on females can be found in Schulze and Musch (Schulze and Musch, 2023). Finally, the use of MCT to produce PH is a limitation and the possibility of MCT-induced toxicity to the diaphragm should be considered when interpreting these findings. Notwithstanding this concern, MCT is a common and effective PH model, eliciting reliable hallmarks of disease such as pulmonary vascular remodeling and subsequent RV hypertrophy (Gomez Arroyo et al., 2012), and has been widely used to study mechanistically the diaphragm in PH (Ahn et al., 2013; de Man et al., 2011; Himori et al., 2017; Kanj et al., 1999; Manders et al., 2012). While off-target effects of MCT on the diaphragm have not been demonstrated, the primary target of MCT given the nature of its metabolism is the pulmonary vasculature and, to our knowledge, there is no evidence that MCT affects skeletal muscle or peripheral vascular function directly. Nonetheless, as with all animal models, application to PH patients should be employed with caution.

### 4.5 Ramifications

The potential consequences of our findings in MCT-induced female rats are that blood flow redistribution toward the ventral costal diaphragm and lack of an increase in medial costal perfusion may be an indication of higher work of a less efficient region of the diaphragm at rest, which would be exacerbated during exercise. This provides a possible mechanism for diaphragm contractile dysfunction as well as dyspnea. Furthermore, the impaired medial costal hyperemic response may also represent an insufficiency to augment blood flow during exercise to this region, which serves as the primary driver of respiration. Importantly, this could contribute to the exercise intolerance reported in PH (Frost et al., 2019). An inability to comfortably be physically active would act to discourage patients from participating in exercise, which has been shown to mitigate symptoms of PH and improve exercise capacity, muscle function, and quality of life in PH (Arena et al., 2015; Becker et al., 2022; Brown et al., 2017; Chan et al., 2013; de Man et al., 2009; Grunig et al., 2011; Mereles et al., 2006). The regional diaphragm blood flow redistribution in the present investigation demonstrates a possible impediment to exercise tolerance in PH, and, notably, exercise training and/or respiratory muscle training may serve as a potential therapeutic approach to improve vascular function and normally distribute diaphragm blood flow, mitigating contractile dysfunction in PH.

### 4.6 Conclusion

**FIGURE 5:**
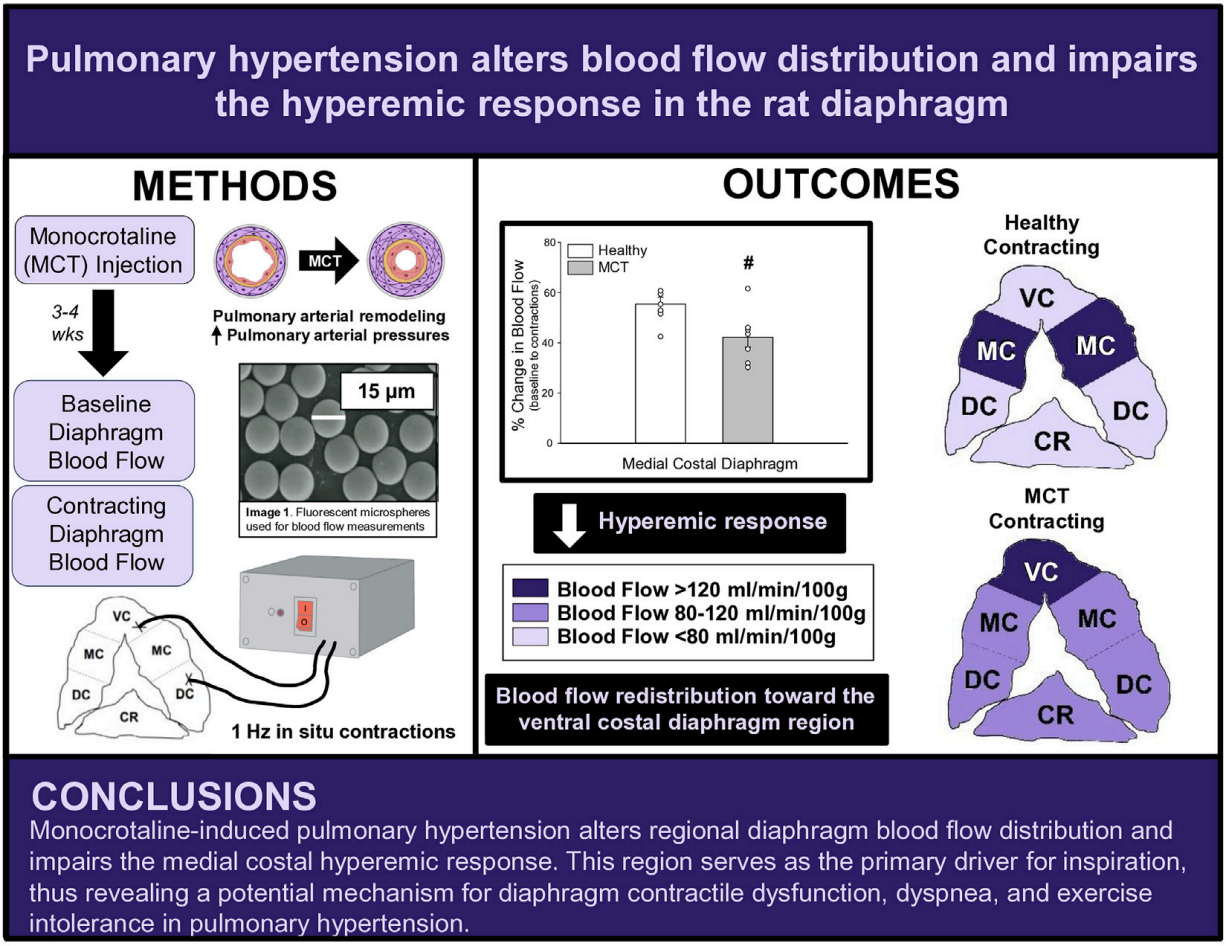
Summary Schematic. VC, ventral costal; MC, medial costal; DC, dorsal costal; CR, crural.

This investigation reports, for the first time, bulk and regional blood flow and the hyperemic response in the diaphragm of PH rats. Our findings demonstrate that PH alters regional diaphragm blood flow distribution and impairs the ability of the medial costal diaphragm to augment blood flow with contractions (Figure 5). Together, these data provide a putative mechanism for PH-induced diaphragm contractile dysfunction. Given that such alterations occurred prior to development of gross diaphragm atrophy, this suggests the presence of vascular dysfunction within the diaphragm which may precede diaphragm fiber weakness. Whether vascular mechanisms associated with the reduced hyperemic response and redistribution of regional diaphragm blood flow are functional and/or structural in nature warrants further scientific investigation.

## Data Availability

The raw data supporting the conclusion of this article will be made available by the authors, without undue reservation.
